# Congenital Disorders of the Human Urinary Tract: Recent Insights From Genetic and Molecular Studies

**DOI:** 10.3389/fped.2019.00136

**Published:** 2019-04-11

**Authors:** Adrian S. Woolf, Filipa M. Lopes, Parisa Ranjzad, Neil A. Roberts

**Affiliations:** ^1^Division of Cell Matrix Biology and Regenerative Medicine, Faculty of Biology Medicine and Health, School of Biological Sciences, University of Manchester, Manchester, United Kingdom; ^2^Royal Manchester Children's Hospital, Manchester University NHS Foundation Trust, Manchester Academic Health Science Centre, Manchester, United Kingdom

**Keywords:** bladder, hydronephrosis, posterior urethral valves, prune belly syndrome, urofacial syndrome, ureter, vesicoureteric reflux

## Abstract

The urinary tract comprises the renal pelvis, the ureter, the urinary bladder, and the urethra. The tract acts as a functional unit, first propelling urine from the kidney to the bladder, then storing it at low pressure inside the bladder which intermittently and completely voids urine through the urethra. Congenital diseases of these structures can lead to a range of diseases sometimes associated with fetal losses or kidney failure in childhood and later in life. In some of these disorders, parts of the urinary tract are severely malformed. In other cases, the organs appear grossly intact yet they have functional deficits that compromise health. Human studies are beginning to indicate monogenic causes for some of these diseases. Here, the implicated genes can encode smooth muscle, neural or urothelial molecules, or transcription factors that regulate their expression. Furthermore, certain animal models are informative about how such molecules control the development and functional differentiation of the urinary tract. In future, novel therapies, including those based on gene transfer and stem cell technologies, may be used to treat these diseases to complement conventional pharmacological and surgical clinical therapies.

## Introduction

The urinary tract comprises the renal pelvis, the ureter, the urinary bladder and the urethra. The tract acts as a functional unit, first propelling urine from the kidney to the bladder, then storing it at low pressure inside the bladder which intermittently and completely voids urine through the urethra. In fact, it was the anatomist Andreas Vesalius who five centuries ago based on his careful autopsy studies (1) reasoned that the kidney, ureter and bladder form a single functional unit. Prior to this, authorities surmised that the kidneys somehow cleaned the blood but they did not necessarily appreciate their anatomical connection with the lower urinary tract.

Congenital diseases of the urinary tract can lead to a range of devastating diseases sometimes associated with fetal losses or kidney failure in childhood and later in life (2–4). In some of these disorders, parts of the urinary tract are absent, while in other cases the organs are present but are severely malformed. In yet other cases, the organs appear grossly intact yet they have congenital functional deficits that can compromise health. Human studies are beginning to indicate monogenic causes for some of these diseases, as reviewed in the last decade (5). Here, we present an update of several of the implicated genes which encode smooth muscle, neural or urothelial structural, and functional molecules, or master transcription factors that regulate their expression. Furthermore, certain animal models are informative about how such molecules control the development and functional differentiation of the urinary tract, and we have alluded to several of them here.

This review does not focus on the detailed anatomy and developmental biology of the kidney and urinary tract, and for reviews of these aspects the reader is referred to other literature (6–8). Furthermore, our focus on the urinary tract does not cover the genetic bases of human kidney malformations *per se* and for this specific topic the reader is directed to other reviews (3, 9, 10). Lastly, although the term ‘congenital anomalies of the kidney and urinary tract', or CAKUT, might be used to include some of the entities we discuss here, we have elected not to use this term because its all-encompassing remit lacks the focus that is required here on the ureter and bladder. Finally, it is important to recognize that factors that perturb embryonic milieu may also adversely impact on urinary tract development: these include maternal diet, vitamin availability, levels of blood glucose and *in vitro* fertilization: again, these aspects are considered elsewhere (11–14).

Table 1 lists the prevalence of several of the congenital urinary tract diseases that will be discussed, and Table 2 list some of the genes implicated in their pathogenesis.

**Table 1:** Prevalences of human congenital urinary tract diseases.

**Urinary tract disease**	**Prevalence**	**References**
Bladder exstrophy	0.002% births	(15)
Megabladder	0.30–0.06% in first trimester	(16)
Posterior urethral valves	0.01% births	(17)
Primary non-syndromic vesicoureteric reflux	Estimated 1–10% in young children	(18)
Prune belly syndrome	0.004% live births	(19)
Ureteropelvic junction obstruction	Up to 0.4% of newborns	(20–22)
Urofacial syndrome	Prevalence unknown but around 150 postnatal cases reported	(23)

**Table 2:** Genes implicated in congenital disorders of the human urinary tract.

*ACTA2* encoding the smooth muscle contractile protein alpha smooth muscle actin
*ACTG2* encoding the smooth muscle contractile protein γ2-actin
*BNC2* encoding basonuclin 2, a zinc finger containing protein implicated in epithelial maturation
*CHRM3* encoding M3, the main acetylcholine receptor in detrusor smooth muscle
*HNF1B* encoding a transcription factor widely expressed in renal tract epithelia
*HPSE2* encoding heparanase 2, a protein that may modulate growth factor signaling in bladder nerves
*ISL1* encoding a transcription factor that may be involved in formation of the bladder and urethra
*LRIG2* encoding leucine-rich-repeats and immunoglobulin-like-domains 2 that may modulate growth factor signaling in bladder nerves
*MYH11* encoding the smooth muscle contractile protein called myosin heavy chain 11
*MYLK* encoding myosin light chain kinase that modifies myosin in smooth muscle cells
*PAX2* encoding a transcription factor widely expressed in the developing ureter and kidney
*TBX18* encoding a transcription factor that affects morphogenesis of the ureter
*TNXB* encoding an extracellular matrix protein found in the urinary tract
*TSHZ3* encoding a transcription factor that modulates smooth muscle differentiation
*UPKIIIA* encoding a member of the uroplakin family that water-proofs the urothelium

## Genetic Studies of Vesicoureteric Reflux, a Common Urinary Tract Malformation

Certain types of urinary tract malformation are thought to be common, such as primary non-syndromic vesicoureteric reflux (VUR). The term describes the retrograde trajectory of urine from the bladder into the upper urinary tract that is neither caused by bladder outflow obstruction (i.e., “primary”) nor associated with malformations outside the renal tract (i.e., “non-syndromic”). The precise prevalence of this condition is uncertain because large asymptomatic populations have not been screened by cystography. The prevalence, however, has been estimated to be as high as 10% percent in babies and young children (18). Prospective studies have shown that milder degrees of VUR usually spontaneously regress during childhood (24). Families with multiple members affected by VUR are recognized yet may not have been exhaustively investigated by cystography. This makes it challenging to track VUR within families. All these aspects make it challenging to undertake genetic studies in primary VUR. Indeed, numerous studies have undertaken genetic linkage or association analyses and indicated various loci as candidates for primary VUR (25–30). Unfortunately, there has been little inter-study uniformity in the loci. Reasons could be that the studies were underpowered, primary VUR is genetically heterogenous, and its modes of inheritance are varied. The largest published study to date (31), using parametric linkage analysis of 1,062 affected individuals from 460 families under a dominant model, identified a single region on chromosome 10q26. The locus contains 69 genes, yet sequencing them failed to reveal likely pathogenic variants in their coding regions. One explanation could be that the region contains mutations in yet-to-be defined non-coding regulatory regions of genes that direct ureter development. Other studies have pointed to variants in specific genes. Two such studies (32, 33) implicated variants of *ROBO2* in primary VUR, and mutations in this gene do cause ureter malformations in mice (32, 34). Others, however, were unable to confirm the observations in other primary VUR populations (35). A similar story of non-replication (36, 37) applies to variants in the gene called *RET* that encodes a growth factor receptor that drives growth of the embryonic ureter rudiment (38). The uroplakins are a family of proteins that form heterodimers that coat the apical surface of the urothelium that lines the renal pelvis, the ureter and the bladder (39). They are thought to confer water-proofing properties and so prevent the egress of urine back into the body. Mice with homozygous mutations of *uroplakin IIIa* (UpkIIIA) or *UpkII* have severe urinary tract malformations including VUR and hydronephrosis (39), and when the zebrafish homolog called upkIII was experimentally knocked-down, embryonic kidney tubule epithelia showed mislocalisation of proteins including the Na^+^/K^+^-ATPase (40). Rare individuals with heterozygous predicted pathogenic *UPKIIIA* mutations have been reported (41–43) and some have ureter malformations, including VUR. In contrast, when large populations with primary non-syndromic VUR were studied, *UPK* mutations could not be identified (44). One explanation could be that *UPK* mutations are rarely compatible with life and so would not be expected to be found in generally healthy individuals with VUR.

## Genetic Breakthroughs in Syndromic Congenital Disorders of the Human Urinary Tract

If discovering specific genetic causes of primary non-syndromic VUR has so far proved elusive, much more progress has been made in defining causative genes in syndromic congenital disorders of the urinary tract i.e., those where there is associated disease outside the urinary tract. In this respect, perhaps the most convincing gene implicated thus far in human primary VUR is *TNXB* that encodes an extracellular matrix protein that is expressed in the urinary tract (45). A heterozygous variant was found to track with VUR in a large family, and cell biology experiments showed that the variant altered cell motility in cultured fibroblasts. Furthermore, rare *TNBX* missense variants were found in certain VUR families in another study (46). Close inspection of affected individuals revealed that some have joint hypermobility and, of note, *TNBX* loss of function mutations had previously been reported in Ehlers-Danlos syndrome (47). Thus, these families with VUR and *TNXB* variants might be considered to have a forme-fruste on the full-blown Ehlers-Danlos syndrome that has been reported to be associated with urinary bladder diverticula (48). Similar observations have been made for *PAX2*, a gene that encodes a transcription factor which is widely expressed in the developing kidney and the urinary tract, and also the optic nerve. Mice with heterozygous mutations of *Pax2* have congenitally small kidneys and can have VUR (49, 50). Rare individuals born with malformed kidneys, sometimes accompanied by VUR, have been found to carry heterozygous *PAX2* mutations (51, 52). A subset of such individuals also have optic nerve malformations (51), so fulfilling all the criteria of the full-blown ‘renal coloboma syndrome'. Again, however, when *PAX2* was sequenced in larger populations with primary non syndromic VUR, mutations could not be identified (53). In the remainder of this review, we will focus on several other rare diseases with defined genetic causes.

## Prune Belly Syndrome

Prune belly syndrome describes a constellation of signs present from the antenatal period featuring a massively distended bladder that fails to empty fully, and overlying abdominal wall muscles that are thinner than normal (54). It nearly always occurs in boys when the signs include undescended testicles. For this condition, the term “sequence” may be more accurate than “syndrome” because the abdominal features could be secondary to the massive bladder distention. The prevalence of prune belly syndrome has been calculated as being 3.8 per 100,000 live births (19). Before birth, the syndrome is one cause of “megabladder,” a massively dilated bladder, a phenotype with a first trimester prevalence of 1:330–1670 (16). From review of the detailed phenotypes (55, 56), prune belly syndrome is likely to have more than one etiology. On histology, some affected individuals have physical blockade of the urethra, for example with urethral agenesis or valves, with a hypertrophied bladder wall. Others, by contrast, have an overtly patent urethra, sometimes with a poorly developed prostatic bed and their bladders can contain disorganized muscle bundles and prominent connective tissue. These two categories would therefore, respectively have either “anatomic” or “functional” bladder obstruction. Contraction of detrusor smooth muscle in the body of the bladder is the driver for urinary voiding, and the neural signal to contract is acetylcholine that is released by parasympathetic autonomic nerves and which binds to the M3 muscarinic receptor. Homozygous, putative loss-of-function mutations, of *CHRM3* have been reported in a family with several males affected by a prune belly-like disease (57). *CHRM3* codes for M3 and the large floppy bladders in this family strikingly resemble the phenotype reported in male homozygous *Chrm3* mutant mice (58). M3 also mediates pupillary contraction to light and the above family showed defects in this reflex (57). A family with a phenotypically similar bladder and eye syndrome have been described but genetic tests were reported to be unrevealing (59). Of note, M3 is present in the embryonic bladder urothelium, as well as in smooth muscle, indicating that it may have other, as yet undefined, roles in the developing urinary tract (57).

Other individuals found to have massively dilated bladders yet no anatomical outflow obstruction carry mutations of genes that encode smooth muscle contractile proteins, or other molecules needed for their functionality. Indeed, mutations of the following genes have been identified in either prune belly or the overlapping disease called megacystis microcolon intestinal hypoperistalsis syndrome: *ACTA2*, encoding α-smooth muscle actin (60); *ACTG2*, encoding γ2 smooth muscle actin (61); *MYH11*, encoding a smooth muscle heavy chain protein (62); and *MYLK* encoding myosin light chain kinase that modifies myosin chains in smooth muscle cells (63). Another candidate gene in relation to myogenic failure is *MYOCD* that encodes a transcription-related protein needed for expression of smooth muscle contractile proteins (64–66). A study that used microarrays to seek copy number variants in 34 cases of prune belly syndrome found that one carried a deletion of a locus encompassing *MYOCD* (67). Finally, heterozygous whole gene deletions of *HNF1B* have been reported in rare patients with prune belly syndrome (68). The gene codes for hepatocyte nuclear factor 1B transcription factor that is widely expressed in epithelia in the developing renal tract (69). On the other hand, while heterozygous mutations of *HNF1B* are well-recognized to cause diverse kidney malformation, these patients do not have prune belly syndrome (70). Moreover, another study found that, while a patient with prune belly syndrome carried a missense variant of *HNF1B*, this did not affect the transactivation functional of the encoded protein (71). A further caveat is that whole gene deletions of *HNF1B* may also extend to adjacent genes (68) so that the final phenotype may not be ascribed to *HNF1B* itself.

## Urofacial Syndrome

Urofacial, or Ochoa, syndrome (UFS) is characterized by a bladder in which the detrusor contracts against an incompletely dilated bladder outflow tract, with the result being high hydrostatic pressures within the bladder yet incomplete voiding (23). It is therefore another example of functional bladder outlet obstruction. Complications include high pressure VUR, ascending bacterial infection, pyelonephritis and renal failure. Affected individuals also have a characteristic grimace when smiling, and a neurogenic basis for this bladder disease has long been surmised (72). The prevalence of the syndrome is not known but at around 150 cases, mostly children, have been reported in the medical literature (23). Inherited in an autosomal dominant manner, a subset of affected families have biallelic mutations of *HPSE2* (73–77) encoding a protein called heparanase 2 that inhibits the enzymatic activity of heparanase (78) that can release growth factors sequestered on matrix molecules called glycosaminoglycans. Other families with UFS instead have biallelic mutations of *LRIG2* (79–81), that encodes a putative plasma membrane protein called leucine-rich-repeats and immunoglobulin-like-domains 2. This protein is thought to mediate growth factor signaling (82), in part by analogy to other better studied LRIG family members (83). Experimental knockdown of the Xenopus hpse2 homolog in embryos causes disorganized peripheral motor nerves (84) and, in mouse developing urinary tracts, both heparanase 2 and LRIG2 can be immunodetected in pelvic ganglia, the structures that send autonomic nerves into the bladder (75, 81). Moreover, both proteins can be detected in nerves growing into normal human fetal bladders (79). Mice that have homozygous mutations for either *Hpse2* (75, 85) or *Lrig2* (81) have impaired bladder emptying. Moreover, these mice have abnormal patterns of bladder nerves, with a depletion around the outflow tract, and an overabundance in the body of the bladder (79). Both mutants also show downregulated levels of bladder transcripts encoding neuronal nitric oxide synthase (81), a protein known to mediate bladder outflow dilatation (86). Thus, evidence is accumulating that UFS is a peripheral neuropathy of the urinary bladder, and it is hoped that the definition of aberrant neurobiology will suggest logical therapies for this devastating disease.

## Congenital Hydronephrosis Caused by Primary Ureter Malformations

Molecularly upstream of myocardin is another transcription factor called teashirt-3, encoded by *TSHZ3* (64, 65, 87). TSHZ3 is normally expressed in mesenchymal cells at the top of the embryonic ureter that are differentiating into smooth muscle cells (64, 88). Homozygous mutant *Tshz3* mice fail to develop ureteric muscle in this location and, unable to peristalsis in this location, have hydronephrotic functionally obstructed ureters (64). Of note, humans with heterozygous deletions of *TSHZ3* can be born with malformed ureters, and these individuals also suffer from an autism-like disorder because the same gene is expressed in, and drives the functional differentiation of, brain cortical neurons (89). Other transcription factors regulate smooth muscle differentiation in the ureter. A preliminary study suggested that TSHZ3 interacts with SOX9 (87) and in humans mutations of the latter gene cause a multiorgan malformation syndrome called campomelic dysplasia (90) featuring sex reversal and hydroureter (91). Similarly, TBX18 is a transcription factor transiently expressed in mesenchymal cells of the embryonic ureter around the urothelium (92). Homozygous mutant *Tbx18* mice form fibroblast-like cells rather than normal smooth muscle in this location (92). Humans with heterozygous *TBX18* pathogenic variants have been reported (93) who have ureteropelvic junction obstruction and hydronephrosis, a phenotype with a postnatal prevalence of up to 0.3–0.4% newborns (20–22). Based on cell culture experiments, it is also possible that the same gene is required to differentiation of pacemaker cells in the ureter (94).

## Posterior Urethral Valves

Posterior urethral valves, a disease confined to males, is a common cause of lower urinary tract obstruction detected by antenatal ultrasound screening. When accompanied by oligohydramnios and kidney damage, neonatal survival is compromised and not significantly improved by fetal vesicoamniotic shunting (4). The prevelance of posterior urethral valves has been calculated to be 1 in 7,800 live births (17). Although generally a sporadic disease, families have been reported with more than one sibling affected (95). Moreover, monozygotic vs. dizygotic twin studies are consistent with a genetic component (96). Currently, however, putative genetic bases for posterior urethral valves remain elusive. Two studies (97, 98) have reported on a variety of copy number variants in patients with posterior urethral valves but a convincing pattern or stronger evidence of pathogenicity yet to emerge for these. Of note, a preliminary study reported multiple members over three generations affected by anatomical uretral obstruction who carried a heterozygous non-sense mutation of *BNC2* (99). This gene codes for basonuclin 2 a zinc finger protein that is expressed in the embryonic urethra (99). Furthermore, experimental downregulation of the homologous gene in zebrafish caused malformation of the distal part of the embryonic urinary tract (99), and mutant *Bnc2* mice have malformed urethras (100).

## Bladder Exstrophy

In classic bladder extrophy, the front part of the bladder is open, and this is considered an intermediate severity disorder in the spectrum of mid-line diseases that span epispadias and cloacal exstrophy. The prevalence has been measured as 2.1 per 100,000 births, with a positive correlation with maternal age (15). Genetic association studies have implicated variation in the *ISL1* locus in classic bladder extrophy, yet pathogenic variants in the *ISL1* coding region have not been proven (101, 102). Another idea is that the association with the ISL1 locus indicates functional variation of a non-coding genomic region that affects ISL1 expression but this hypothesis has yet to be proven (103). *ISL1* is a transcription factor known to be expressed in the region of the forming mouse bladder (102). Mutant *Isl1* mice have an epispadias-like phenotype, and in normal development the encoded transcription factor induces bone morphogenetic factor 4 (BMP4) mediated remodeling of mesenchymal cells (104). Of note, BMP4 is also immunodetected in the walls of human embryonic bladders (105) and other experiments suggest its expression is under the control of sonic hedgehog, a growth factor secreted by embryonic urothelium (65, 105). Several studies have sought copy number variants in the bladder extrophy-epispadias spectrum, and a small but statistically significant subset of affected individuals have duplication of chromosome 22q11.2 (106–108). The critical region contains numerous genes expressed in the developing kidney and urinary tract including *CRKL*, encoding a transforming protein kinase, that itself has been implicated as causing the kidney malformation found in DiGeorge syndome (109). In this context, however, the gene dosage is reduced (i.e., “haploinsufficiency”) rather than amplified and, moreover, the DiGeorge syndrome does not feature bladder extrophy.

## Conclusions and Novel Therapeutic Perspectives

As discussed above, the genetic breakthroughs in this field of human disease have come from investigating families with rare congenital urinary tract diseases. Here, studies are indicating that the implicated genes encode smooth muscle, neural or urothelial molecules, or master transcription factors that regulate their expression. To date, however, variants in these same genes do not appear to explain the more common human non-syndromic urinary tract malformations such as primary VUR. Whole exome sequencing, a technology that seeks variants in the protein coding regions of all genes, is being applied to seek likely pathogenic mutation in clinical cohorts, including adult with chronic kidney disease (110) and children born with a range kidney malformations (111). Such research exercises have yielded useful genetic information in 10–14% of cases tested.

In future, it may be informative to apply these technologies to groups of patients born with the urinary tract malformations described in the current review. It is important to remember, however, that mutations may not be the only explanation for congenital urinary tract malformations. As alluded to in the *Introduction*, the fetal environment can be modified by alterations in maternal diet or the presence of maternal disease, such as diabetes mellitus (11–14). These non-genetic changes might themselves perturb the normal trajectory of organogenesis. Along the same lines, epigenetic alternations, such as DNA methylation, may profoundly affect expression of renal genes and thus impact on the propensity to disease. For example, such a mechanism has recently been implicated in gene expression of aging human kidneys (112) and there exists preliminary evidence that similar mechanisms may be operative in human renal malformations (113).

At least for the rare syndromes discussed in this review, as the genetics and pathobiology becomes defined, it is possible to begin to envisage smart biological therapies for these diseases to complement conventional pharmacological and surgical clinical interventions. Indeed, gene therapy, for example mediated by viral vectors, may offer promise. In a recent striking breakthrough, babies with spinal muscular atrophy 1 caused by *survival motor neuron 1* mutations were intravenously administered adeno-associated virus (AAV) that transduced *SMN1*, the gene that is at fault in this disease. By 20 months the infants were alive and developing well, whereas untreated individuals would have been expected to have died or alive but severely paralyzed (114). The AAV viral vector used is a non-pathogenic virus that rarely integrates into the host genome, and numerous trials with AAV vectors have been registered for monogenic and other disorders. Of note, specific AAV serotypes can deliver reporter genes into developing mouse renal tracts after maternal, embryonic and neonatal administration; proven targets include kidneys (AAV9), urinary bladders (AAV9) and autonomic and dorsal root and gut ganglia (AAV8 and 9) (115–118). The urinary tract diseases with defined genetic bases, described in this review, meet key criteria for being suitable to be treated with viral gene delivery. Current management for these diseases only attempts to control symptoms, for example with physical bladder drainage, rather than cure the disease process. The diseases are genetically defined, with well-defined links between the normally encoded proteins and disease phenotypes. Moreover, there are phenotypically faithful genetic mouse models on which to test novel biological treatments as first steps to the clinic. The selection of patients who may benefit from novel therapies would, however, have to be highly judicious because the presence of a mutant gene may not in itself be sufficient to generate a clinically significant malformation. This is because the severity of disease in an individual may theoretically be altered by as-yet poorly defined modifying genes, epigenetic changes, and alterations of the fetal environment.

## Author Contributions

All authors listed have made a substantial, direct and intellectual contribution to the work, and approved it for publication.

### Conflict of Interest Statement

The authors declare that the research was conducted in the absence of any commercial or financial relationships that could be construed as a potential conflict of interest.
